# Combined Brain-Heart Magnetic Resonance Imaging in Autoimmune Rheumatic Disease Patients with Cardiac Symptoms: Hypothesis Generating Insights from a Cross-Sectional Study

**DOI:** 10.3390/jcm9020447

**Published:** 2020-02-06

**Authors:** George Markousis-Mavrogenis, Dimos D. Mitsikostas, Loukia Koutsogeorgopoulou, Theodoros Dimitroulas, Gikas Katsifis, Panayiotis Argyriou, Dimitrios Apostolou, Stella Velitsista, Vasiliki Vartela, Dionysia Manolopoulou, Maria G. Tektonidou, Genovefa Kolovou, George D. Kitas, Petros P. Sfikakis, Sophie I. Mavrogeni

**Affiliations:** 1 Onassis Cardiac Surgery Center, 17674 Athens, Greece; georgemm32@gmail.com (G.M.-M.); vasvartela@yahoo.gr (V.V.); dmanolopoulou@yahoo.com (D.M.); genovefa@kolovou.com (G.K.); 2 First Neurology Department, National and Kapodistrian University of Athens, 10679 Athens, Greece; dimosmitsikostas@me.com; 3 Department of Pathophysiology, Laikon Hospital, 11527 Athens, Greece; lukia.km@gmail.com; 4 Department of Rheumatology, Aristotle University of Thessaloniki, 54124 Thessaloniki, Greece; dimitroul@gmail.com; 5 Rheumatology Department, Naval Hospital, 11521 Athens, Greece; katsifisg@yahoo.gr; 6 MRI Unit, Mediterraneo Hospital, 16675 Athens, Greece; pan_argyriou@yahoo.gr (P.A.); apostolou@mediterraneohospital.gr (D.A.); svelitsista74@gmail.com (S.V.); 7 First Department of Propaedeutic and Internal Medicine, Laikon Hospital, National and Kapodistrian University of Athens, 11527 Athens, Greece; mtektonidou@gmail.com (M.G.T.); psfikakis@med.uoa.gr (P.P.S.); 8 Arthritis Research UK Epidemiology Unit, Manchester University, Manchester M13 9PT, UK; gkitas@hygeia.gr

**Keywords:** vasculitis, brain lesion, white matter hyperintensity, cardiovascular disease, neuro-psychiatric symptoms, cognitive dysfunction, brain–heart interaction

## Abstract

Background: Autoimmune rheumatic diseases (ARDs) may affect both the heart and the brain. However, little is known about the interaction between these organs in ARD patients. We asked whether brain lesions are more frequent in ARD patients with cardiac symptoms compared with non-ARD patients with cardiovascular disease (CVD). Methods: 57 ARD patients with mean age of 48 ± 13 years presenting with shortness of breath, chest pain, and/or palpitations, and 30 age-matched disease-controls with non-autoimmune CVD, were evaluated using combined brain–heart magnetic resonance imaging (MRI) in a 1.5T system. Results: 52 (91%) ARD patients and 16 (53%) controls had white matter hyperintensities (*p* < 0.001) in at least one brain area (subcortical/deep/periventricular white matter, basal ganglia, pons, brainstem, or mesial temporal lobe). Only the frequency and number of subcortical and deep white matter lesions were significantly greater in ARD patients (*p* < 0.001 and 0.014, respectively). ARD vs. control status was the only independent predictor of having any brain lesion. Specifically for deep white matter lesions, each increase in ECV independently predicted a higher number of lesions [odds ratio (95% confidence interval): 1.16 (1.01–1.33), *p* = 0.031] in ordered logistic regression. Penalized logistic regression selected only ARD vs. control status as the most important feature for predicting whether brain lesions were present on brain MRI (odds ratio: 5.46, marginal false discovery rate = 0.011). Conclusions: Subclinical brain involvement was highly prevalent in this cohort of ARD patients and was mostly independent of the severity of cardiac involvement. However, further research is required to determine the clinical relevance of these findings.

## 1. Introduction

In recent years, a paradigm of recognizing the interactions between cardiac pathologies and other organ systems has gradually developed. In this context, entities in the form of the cardiorenal and cardiohepatic syndromes [1], as well as the important interplay between cardiovascular disease and the immune system [2] have attracted significant scientific attention. The next logical step in this paradigm is the interaction between the brain and the heart in health and disease. These two organs share significant anatomical similarity in their vascular anatomy and organization, with both relying on deep penetrating arteries arising from conduit arteries on their surface for tissue perfusion. Small vessel disease (SVD) can manifest in the penetrating vessels of both organs, leading to dysfunction of their respective perfusion domains [3,4,5]. Current evidence also supports the notion that cardiac pathologies may set the stage for the development of pathologic alterations of the cerebral parenchyma. The prototypical example of this is atrial fibrillation (AF); in a recent study, more than 1/5 AF patients had cortical/non-cortical stroke or microbleeds and almost all AF patients had white matter hyperintensities (WMHs), despite being appropriately treated with anti-coagulation [6].

Importantly, neurologic deficits or cognitive dysfunction may not become apparent for considerable time intervals after the appearance of cerebral parenchymal lesions [7,8,9]. Among subclinical cerebral alterations, WMHs are of particular clinical interest. They are defined as patchy areas of signal hyperintensity and are evident on brain magnetic resonance imaging (MRI) T2-weighted or fluid attenuation inversion recovery (FLAIR) sequences [9]. Approximately 11–21% of healthy subjects with mean age of 64 years and 64–94% of those with mean age of 82 years are affected by WMHs [7,8,9,10], and their presence is associated with reduced ability to carry out daily activities, gait abnormalities, mood disturbances and a high risk of stroke, dementia and death [9].

Autoimmune rheumatic diseases (ARDs) are a heterogeneous group of diseases resulting from immune dysregulation. With improvements in immunosuppressive treatment modalities, cardiovascular disease (CVD) has emerged as the most frequent cause leading to higher mortality in these patients [11]. In recent years, a concerted effort has been made to improve the diagnosis and treatment of CVD in ARD patients [12], leading some to coin the term “cardiorheumatology” to describe this nascent branch of cardiology [13]. The driving force behind CVD in ARD patients is systemic inflammation [11], yet, even though similar mechanisms may drive neuroinflammation and subsequent damage of the brain parenchyma [14,15], very little is currently known regarding the interaction between heart and brain involvement in ARD patients. 

MRI is the diagnostic modality of choice for the cardiac evaluation of ARD patients [12] and is the only imaging modality that can also adequately visualize the brain parenchyma in a single examination. A large volume of literature has shown that various non-autoimmune CVDs may lead to the development of WMHs [16,17,18,19,20]. Additionally, according to a systematic review by Wiseman et al., the risk of stroke is higher in ARD patients compared with the general population [21]. In particular, patients <50 years old, with rheumatoid arthritis or systemic lupus erythematosus may experience ischemic and hemorrhagic stroke risk increases of 60% up to 100% [21]. It is thus known that CVD in general is linked with WMHs and that ARD patients tend to have a higher prevalence of brain involvement. However, whether the presence of WMHs is at all related with the severity of cardiac involvement has not been previously studied. The primary aim of this study was thus to perform a combined heart/brain MRI study of ARD patients presenting with cardiac symptoms and matched disease controls with non-autoimmune CVD, in order to compare the prevalence of WMHs in both groups and to investigate potential associations between WMH burden and the severity of cardiac dysfunction based on MRI indices.

## 2. Patients Methods

### 2.1. Patients

Patients with known ARD diagnosis and symptoms of chest pain, palpitations and/or shortness of breath, were prospectively recruited after being referred to our tertiary center for cardiovascular evaluation. All patients were consecutive and were included over a period of 2 years (2017–2019). A complete cardiovascular workup including standard clinical examination, echocardiography and imaging with cardiovascular magnetic resonance (CMR) was performed on all recruited patients. No recent or past evidence of pulmonary hypertension or embolism was identified in any patients. In addition, all patients underwent a comprehensive clinical neurologic evaluation to exclude dementia, memory loss and/or cognitive dysfunction and a complete brain MRI was performed immediately following CMR evaluation. Thirty age-matched patients with cardiac symptoms but without any clinical evidence of brain involvement and no known ARD diagnosis were also recruited as disease-controls and underwent the same evaluations as ARD patients. All patients that underwent MRI scans were included in the final analysis. Exclusion criteria for this study were potential contraindications to MRI (non-MRI conditional metal prosthetics or devices, known allergy to paramagnetic gadolinium-based contrast agents, pregnancy or renal failure) and clinical evidence of dementia, memory loss and/or cognitive dysfunction, which none of the initially recruited patients met. Collectively no patients had any objectifiable abnormalities in either their neurologic or cardiac clinical evaluations, and standard echocardiography did not demonstrate cardiac dysfunction. All patients signed a written informed consent form and the study protocol was approved by the Onassis Cardiac Surgery Center medical ethics committee (13/4/2015; identification: Mavrogeni-ARD1) before implementation.

### 2.2. Methods

All MRI investigations were performed on a 1.5-T scanner (Ingenia, Philips Medical Systems, Eindhoven, The Netherlands).

### 2.3. CMR Study

The CMR protocol included standard steady-state free-precession cine CMR, black-blood T2-weighted short tau inversion recovery images, T1-weighted spin-echo early gadolinium enhancement (EGE) CMR, and phase-sensitive inversion recovery late gadolinium enhancement (LGE) CMR as described previously [6]. A dose of 0.1 mmol/kg gadobenate dimeglumine contrast-medium was injected for the acquisition of EGE and another 0.1 mmol/kg for the acquisition of LGE images, as suggested by the Lake Louise criteria [22]. T1-mapping was performed using a modified Look-Locker inversion recovery (MOLLI) sequence with a 3(3)5 scheme on 3 representative short-axis positions before and 15 minutes after contrast-medium administration. T2-mapping was performed on 3 corresponding LV short-axis slices using a black-blood prepared, navigator-gated, free-breathing hybrid gradient (echo planar imaging), and spin-echo multiecho sequence [23].

### 2.4. CMR Data Analysis

Global myocardial inflammation was assessed based on T2-weighted images by calculating the T2 signal intensity ratio as signal intensity of myocardium divided by signal intensity skeletal muscle [22]. Global relative enhancement was calculated by measuring myocardial signal intensity on pre- and post-contrast T1-weighted spin-echo images relative to skeletal muscle [22]. The presence and pattern of non-ischemic LGE lesions were qualitatively assessed by consensus agreement of 2 experienced observers. Native and post-contrast T1-, ECV, and T2-maps were generated [12]. Global native/post-contrast myocardial T1, ECV, and T2 values were calculated as the mean value of 3 short-axis slices. The accuracy of the T1- and T2-mapping methods was validated according to previously described protocol [23].

### 2.5. Brain MRI Study

A standard brain MRI protocol for CV evaluation was followed, which included the following measurements [24]:

(a) Spin-echo T1- and T2-weighted imaging,
(b) fluid-attenuated inversion recovery (FLAIR) imaging,
(c) diffusion-weighted (DW) imaging,
(d) susceptibility-weighted (SW) imaging,
(e) time-of-flight (TOF) MR angiography, and
(f) contrast-enhanced T1-weighted imaging (using fat sat suppression and flow compensation) and T1-weighted images (3 mm or less) of areas of abnormality on MRA/TOF.

### 2.6. Statistical Analysis

Statistical analyses were carried out using the software Stata v.15SE (College Station, TX: StataCorp LLC.) and R version 3.6.1 (RStudio, Inc., Boston, MA). Normality of continuous variables was evaluated visually using Q-Q plots and/or histograms. Normally distributed variables were compared between ARD patients and disease controls using independent sample t-tests, continuous not-normally distributed variables were compared using Mann–Whitney tests and categorical/binary variables were compared using chi-square tests or Fisher’s exact tests if appropriate. Additional univariable pooled univariable analyses were performed between patients of any group with and without identified brain lesions. Normally distributed variables are presented as mean (standard deviation), continuous not-normally distributed variables are presented as median (interquartile range) and categorical/binary variables are presented as number (%).

### 2.7. Regression Analyses

The presence and number of identified brain lesions were used as outcomes in pooled regression analyses of the ARD and control groups. The association of the presence of any brain lesions with CMR indices and which group the patient belonged to was examined for each variable separately using univariable logistic regression analysis and multivariable logistic regression analysis adjusted for age and sex. The number of brain lesions at the most frequently affected brain sites were also examined for association with the same variables using univariable and multivariable ordered logistic regression analysis. All resulting p-values were corrected for multiple testing using the Benjamini–Hochberg correction, to ensure that no spurious associations are identified due to inflated type-I error. 

### 2.8. Feature Selection

The ncvreg package for R [25] was used to carry out minmax concave penalty (MCP) logistic regression analyses with k-fold cross-validation for discriminating between patients with and without brain lesions. All CMR indices as well as ARD vs. control status were investigated as potential features. The optimal value for the penalization term λ was determined as the value that minimizes the cross-validation error rate derived from k-fold cross-validation. The reliability of selected features was evaluated using the built-in marginal false discovery rate (mFDR), which performs better than other inference methods for penalized regression analyses [26,27]. Model predictive capacities are reported as cross-validated R2 values. Penalized regression analysis can overcome the disadvantages of stepwise or best subset approaches for feature selection [28] and allows for the selection of important predictors by optimizing the variance-bias tradeoff [29]. This ensures optimal external validity for the identified predictors at the cost of more biased estimates. The employed type of penalization (MCP) has been shown to be less biased towards features with larger coefficients than other penalization methods like least absolute shrinkage and selection operator (LASSO) [25,28] and was thus preferred.

## 3. Results

This study finally included 57 ARD patients and 30 disease controls. The prevalence of specific diseases/syndromes in each group is presented in detail in Table 1. The baseline characteristics of ARD patients and disease controls are also presented and compared with each other in Table 1. ARD patients were mostly of female sex [40 (70%) vs. 7 (23%), *p* < 0.001] and only differed in cardiac function with regard to RVEF which was higher compared with controls [64.0 (60.0–68.0) vs. 60.5 (55.0–65.0), *p* = 0.031]. With regard to tissue characterization indices, only post-contrast T1-mapping and ECV differed significantly between the two groups. ECV was namely significantly higher in ARD patients compared with controls [29.0 (28.0–32.0) vs. 26.0 (25.0–28.0), *p* < 0.001]. Similarly, the proportion of patients with pathologic ECV values was higher in ARD patients. The same was the case for the proportion of ARD patients with pathologic T2-mapping values [25 (44%) vs. 5 (17%), *p* = 0.011]. No other tissue characterization indices differed between the two groups. Of the 57 ARD patients, 34 (58.6%) were 50 years old or younger.

**Figure 1:**
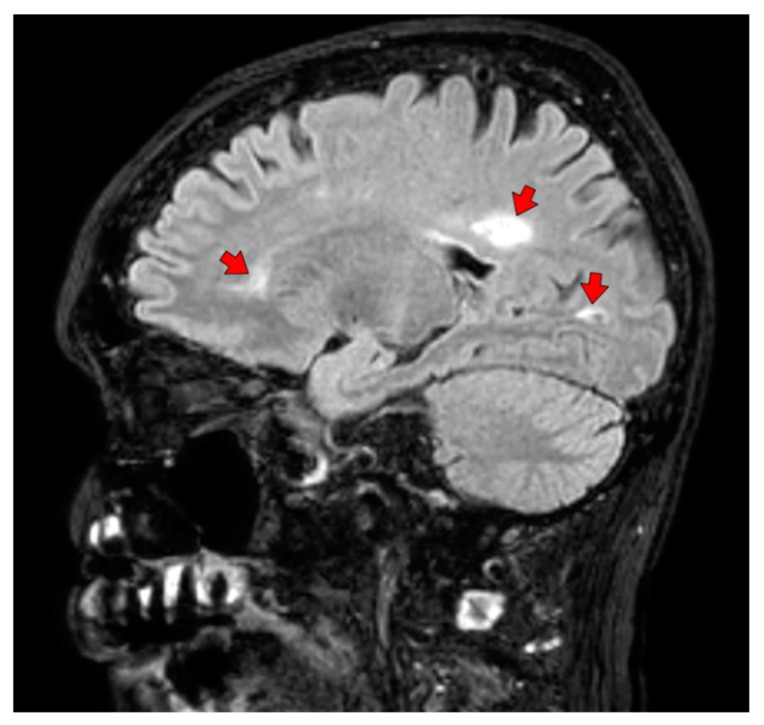
A fluid attenuation inversion recovery (FLAIR) image from a patient with sarcoidosis showing silent white matter hyperintensities.

Brain lesions were identified only in FLAIR images (Figure 1). Overall, ARD patient were significantly more likely to have at least one brain location with lesions identified by MRI [52 (91%) vs. 16 (53%), *p* < 0.001]. However, all ARD patients with brain lesions had one or more lesions in a single brain area, while some disease controls with brain lesions had 2 or 3 different affected brain areas (*p* < 0.001). Brain lesions were identified in both groups in numerous locations, including the subcortical, deep and periventricular white matter, the cortex, the basal ganglia, the pons, the brainstem and the mesial (medial) temporal lobe. With the exception of pontine lesions which were only identified in ARD patients and cortical lesions which were only identified in disease controls, all other locations were affected by one or more lesion in patients of both groups. Of the 5 ARD patients without brain lesions, 2 were diagnosed with systemic sclerosis, and 1 each was diagnosed with SLE, RA, and GPA. Only the frequency and number of subcortical and deep white matter WMHs were significantly greater in ARD patients compared with controls (*p* < 0.001 and 0.014 respectively). There was a similar trend for periventricular WMH which did not reach statistical significance (*p* = 0.073). For each of the latter three anatomical locations, univariable and multivariable (corrected for age and sex) ordered logistic regression analyses were performed to identify predictors of local lesion burden (Supplementary Tables S1–S3). ARD vs. control status was an independent significant predictor in all cases. Specifically for deep WMH, each increase in ECV independently predicted a higher number of lesions [odds ratio (95% confidence interval): 1.16 (1.01–1.33), *p* = 0.031].

When comparing the same baseline characteristics between participants with and without brain involvement belonging to any group (Table 2), the proportion of female patients with brain involvement was greater compared with that of patients without brain involvement [43 (63%) vs. 4 (21%), *p* = 0.001]. The proportion of ARD patients with brain lesions was also significantly higher compared to those without [52 (76%) vs. 5 (26%), *p* < 0.001]. Similarly, ECV was significantly higher in patients with brain lesions compared to those without [29.0 (27.0–32.0) vs. 26.0 (25.0–29.0), *p* = 0.002]. The proportion of patients with pathologic ECV and T2-mapping values followed a trend for higher values in patients with brain lesions, but this fell shy of statistical significance (*p* = 0.052 in both cases). No other CMR indices differed significantly between patients with and without brain lesions.

Logistic regression analyses (Table 3) for predicting the presence of any type of lesion did not yield any significant CMR variables in either univariable testing or testing with multivariable correction for age and sex. Only ARD vs. disease control status was significantly associated with a higher probability of having brain lesions both in univariable analysis [odds ratio (95% confidence interval): 9.10 (2.84-29.17), *p* < 0.001] and when adjusted for age and sex [OR (95%CI): 6.26 (1.74–22.49), *p* = 0.005]. A penalized logistic regression analysis for feature selection with regard to the same outcome was performed using the MCP method as described previously in the methods section. Out of all available CMR indices, age, sex and ARD vs. control status, this analysis selected only ARD vs. control status as the most important feature for predicting the probability of having brain lesions (estimated odds ratio: 5.46, mFDR = 0.011). 

## 4. Discussion

In this study, we report for the first time a comparison of the prevalence of WMH in ARD patients and disease controls with CVD. Brain MRI identified occult brain lesions in the vast majority of ARD patients, presenting with cardiac symptoms and approximately half of all disease controls. These were WMHs mainly localized in the subcortical, periventricular, and deep white matter. In contrast to controls, all ARD patients with brain lesions had only one brain area affected. However, ARD patients had a heavier burden of WMHs within each affected area. Cardiac involvement in ARD patients manifested as a non-ischemic fibrotic pattern with/without concurrent myocardial odema. ARD patients had on average higher RVEF and ECV values as well as a greater proportion of pathologic ECV and T2-mapping values compared with controls. Only ARD vs. control status was a significant independent predictor of the occurrence of any brain lesion, as well as of local lesion burden. Higher ECV was also independently associated with a heavier deep white matter lesion burden. 

CMR findings in ARD patients are according to expectations for this population. The higher observed RVEF is secondary to chronic RV involvement often seen in these patients. This can be attributed to the increased RV workload resulting from compromised LV function, inflammation, and myocardial fibrosis inherent to ARDs, combined with pulmonary parenchymal changes, pulmonary hypertension and/or chronic repeated pulmonary embolism [30]. However, it could also be argued that the modest ARD population of this study might not fully represent the entire ARD patient spectrum and might thus account for this finding. Furthermore, a combination of pathologic T2-mapping and ECV is suggestive of myocardial inflammation/fibrosis, the main characteristics of cardiac involvement in ARDs [23,31,32,33].

This investigation clearly demonstrates a significantly higher prevalence of WMHs in ARD patients compared with a disease control group with known predisposition. Importantly, ~60% of all ARD patients were 50 years old or younger. WMHs are also common in individuals with acquired immunodeficiency, such as human immunodeficiency virus (HIV)-positive patients, even in those with low viral loads, and are strongly correlated with reduced gait speed and impaired cognitive function [34]. Individuals with periventricular white matter lesions perform nearly 1 standard deviation (SD) below average on tasks involving psychomotor speed. Deep white matter lesions affect cognition even more severely than periventricular lesions [35] and were also highly prevalent in our ARD population (~60%). Subjects with severe periventricular lesions experienced cognitive decline nearly 3 times faster than average, independent of age, gender, education, depression, and infarcts in a recent study [36]. In general, a leukoaraiosis of 3% is enough to reduce working memory scores by 2 standard deviations [37]. Known factors associated with the occurrence of WMHs in general are a number of vascular risk factors, including large vessel atherosclerosis, SVD and elevated plasma homocystein and serum carotenoid levels [38]. Etiologically, these may mainly result from hypertension, smoking and/or type-2 diabetes mellitus [38]. However, these are probably not the driving force in our cohort, as the prevalence of these risk factors was very low in either group. Thus, collectively, these findings suggest that WMHs may be not benign lesions and the fact that they are highly prevalent even in younger ARD patients should raise concern as to why this issue remains unaddressed.

The importance of these findings becomes even more apparent when taking recent literature into account. Namely, the risk of developing dementia was found to be significantly higher in middle-aged ARD patients compared with the general population [39]. Furthermore, the risk of any type of stroke is higher in ARD patients than in the general population, particularly in those under 50 years. Rheumatoid arthritis and SLE increase ischemic and hemorrhagic stroke risk by 60–100% relative to the general population [21]. Lastly, WMHs in asymptomatic patients with systemic vasculitides suggest the presence of microangiopathy and can lead to cognitive impairment [40,41]. Similarly, cerebral vasculitis was also detected in SLE [42], Behcet disease [43], and neurosarcoidosis, potentially even occurring as isolated involvement [44,45]. 

To our knowledge, this is the first study combining heart and brain magnetic resonance evaluation in ARD patients with cardiac symptoms but no neurologic history/symptoms or known/overt neurologic disease. We have previously reported that occult CMR lesions, including myocardial odema, myocarditis, diffuse subendocardial fibrosis and myocardial infarction are not unusual in treatment naïve ARD patients, and may be reversible after appropriate treatment [46]. Similarly, cardiac lesions, missed by echocardiography, were detected by CMR in SLE patients in another study by our group [47]. The CMR findings in the current ARD cohort are thus in agreement with previous literature by our group and others regarding the spectrum of CMR lesions in ARDs [23,31,32,33]. Interestingly, the only CMR index specifically associated with a heavier burden of deep WMHs was myocardial ECV. Using a primary distinction between deep and periventricular WMHs, deep lesions are more often of ischaemic nature, usually resulting from SVD, while periventricular lesions tend to be non-ischaemic [38]. The high prevalence of deep white matter lesions in ARD patients of this study and the association of ECV with heavier lesion burden thus supports the notion that SVD, which is also common in various ARDs [48], may be constitute the common denominator shared between deep WMHs and cardiac involvement in ARDs. This might also explain why ECV was not associated with lesion burden in other locations with predominantly non- ischaemic lesions. 

A number of studies of population-based cohorts have demonstrated associations between plasma levels of circulating C-reactive protein and interleukin-6 levels and brain SVD [49,50], independent of age and cardiovascular risk factors. Interestingly, with the exception of the association with ECV, the occurrence of WMHs was largely independent of CMR parameters and chiefly related to the diagnosis of ARD vs. CVD. This suggests that the severity of cardiac inflammation and fibrosis is related only to ischaemic and not to non- ischaemic WMH occurrences. As such, this demonstrates the need for a combined brain and heart MRI examination in ARD patients with cardiac symptoms, seeing as disease progression may evolve differently in the two organs. Notably, our group has previously reported that CMR findings are independent of C-reactive protein levels in ARD patients [47,51,52], often rendering biomarker-based pre-selection of patients for CMR examinations problematic. This would explain the current findings and suggests that such inflammatory biomarkers might have greater utility in evaluating brain involvement than cardiac involvement, although this needs to be corroborated by future independent studies.

The findings of this investigation have important implications for the clinical evaluation and management of ARD patients. Next to the known additive value of CMR in the evaluation of ARD patients with cardiovascular symptoms [53], this investigation demonstrates that brain MRI identified WMHs in the vast majority of ARD patients. MRI is the only non-invasive imaging modality that can evaluate both the heart and the brain without employing ionizing radiation. It offers high spatial resolution and the ability to characterize tissues based on their composition, while being operator-independent and highly reproducible. Considering the adverse effect of WMHs on cognition and notably the relatively young age of ARD patients in this study, these findings suggest that a combined brain–heart MRI investigation might be an appropriate diagnostic consideration in these patients. Particularly in the case of SVD, timely initiation of immunomodulatory treatment and aspirin as needed, might attenuate macro- and microvascular damage in ARDs [54]. CMR can play a complementary role in guiding immunomodulatory treatment in ARD patients. However, the appropriate approach to ARD patients with combined heart and brain involvement is largely undefined and no therapeutic guidelines exist to direct therapeutic interventions. Therefore, large multicenter studies with long-term follow up are required to confirm the clinical impact of our findings, to provide long-term prognostic and to investigate potential etiologic treatments.

## 5. Limitations

This investigation was mainly limited by the small sample size and the lack of long term (>5 years) cardiac and neurologic follow-up. Additionally, we report an association between ECV and deep WMH burden. However, this could potentially be confounded by the fact that on average ARD patients had higher ECV values, since collectively only ARD vs. control status independently predicted brain lesion occurrence. The size of our cohort does not permit statistical testing to rule this eventuality out. Lastly, a more comprehensive evaluation of plasma inflammatory biomarker levels was not available and thus no inferences could be made in that regard.

## 6. Conclusions

In this first head-to-head comparison between ARD patients with cardiac symptoms and CVD controls, combined brain–heart MRI identified cardiac abnormalities and small size WMHs in the vast majority of ARD patients, despite the absence of clinical signs/symptoms. The occurrence of brain lesions was largely independent of CMR indices and was related to ARD vs. control status. Thus, ARD patients are at higher risk of developing WMHs compared with matched CVD controls. However, further research is required to confirm these findings and to clarify their clinical importance.

## Figures and Tables

**Table 1:** Characteristics compared between ARD patients and disease controls. * *p* ≤ 0.05.

Variable	ARD Patients	Disease Controls	*p*-Value
**Number of Patients**	57	30	N/A
**Demographics**			0.68
Age	46.9 (13.1)	48.2 (14.9)	<0.001 *
Female Sex	40 (70%)	7 (23%)	
**Type of ARD**			
Systemic Lupus Erythematosus	14 (25%)	N/A	N/A
Systemic Sclerosis	10 (18%)		
Sarcoidosis	5 (9%)		
Granulomatosis with Polyangiitis	5 (9%)		
Ankylosing Spondylitis	5 (9%)		
Eosinophilic Granulomatosis with Polyangiitis	4 (7%)		
Rheumatoid Arthritis	4 (7%)		
Takayasu Arteritis	3 (5%)		
Adamantiades-Behcet Disease	3 (5%)		
Antiphospholipid Syndrome	1 (2%)		
Juvenile Rheumatoid Arthritis	1 (2%)		
Dermatomyositis	1 (2%)		
Enteropathic Arthritis	1 (2%)		
**Type of Cardiovascular Disease**			
Myocarditis	N/A	5 (17%)	N/A
Coronary Artery Disease		5 (17%)	
Duchenne Muscular Dystrophy		3 (10%)	
Hypertension		3 (10%)	
Arrhythmia		2 (7%)	
Non-compaction Cardiomyopathy		2 (7%)	
Atrial Septal Defect		2 (7%)	
Amyloidosis		1 (3%)	
Takotsubo Cardiomyopathy		1 (3%)	
Myotonic Dystrophy		1 (3%)	
Dilated Cardiomyopathy		1 (3%)	
Peripartum Cardiomyopathy		1 (3%)	
Arrhythmogenic Right Ventricular Cardiomyopathy		1 (3%)	
Myopericarditis		1 (3%)	
Myocardial Infarction		1 (3%)	
**Cardiovascular Risk Factors**			
Hypertension	4 (7%)	2 (7%)	0.951
Diabetes Mellitus	2 (4%)	3 (10%)	0.219
Current Smoker	2 (4%)	1 (3%)	0.966
Atrial Fibrillation	0 (0%)	0 (0%)	0.999
**Cardiac Functional Indices**			
LVEDV (mL)	134.0 (109.0, 160.0)	135.5 (105.0, 163.0)	0.63
LVESV (mL)	50.0 (37.0, 63.0)	50.5 (38.0, 66.0)	0.67
LVEF (%)	62.0 (60.0, 68.0)	62.0 (56.0, 69.0)	0.55
RVEDV (mL)	122.0 (97.0, 137.0)	128.0 (110.0, 161.0)	0.13
RVESV (mL)	42.0 (35.0, 53.0)	47.5 (34.0, 64.0)	0.16
RVEF (%)	64.0 (60.0, 68.0)	60.5 (55.0, 65.0)	0.031 *
**Tissue Characterization Indices**			
T2 Signal Ratio	2.0 (1.8, 2.3)	2.0 (1.7, 2.2)	0.7
EGE (%)	2.6 (1.6, 3.2)	2.5 (2.0, 3.0)	0.89
LGE (%)	4.0 (0.0, 5.0)	4.5 (0.0, 6.0)	0.38
Native T1-Mapping (ms)	1048.0 (1005.0, 1084.0)	1034.0 (998.0, 1055.0)	0.33
Post-contrast T1-Mapping (ms)	387.0 (348.0, 410.0)	419.0 (358.0, 435.0)	0.018 *
ECV (%)	29.0 (28.0, 32.0)	26.0 (25.0, 28.0)	<0.001 *
T2-Mapping (ms)	55.4 (7.1)	52.3 (7.7)	0.071
**Local Cut-off Points for CMR Indices**			
LGE > 0%	33 (58%)	21 (70%)	0.27
EGE ≥ 4	13 (23%)	4 (13%)	0.29
T2 Ratio ≥ 2	33 (58%)	18 (60%)	0.85
T2-Mapping > 55 ms	25 (44%)	5 (17%)	0.011 *
Native T1-Mapping > 1050 ms	25 (44%)	10 (33%)	0.34
ECV ≥ 29%	33 (58%)	7 (23%)	0.002 *
Post-contrast T1-Mapping < 350 ms	15 (26%)	4 (13%)	0.16
LVEF < 55%	7 (12%)	6 (20%)	0.34
**Brain MR Findings**			
Brain Involvement (any location)	52 (91%)	16 (53%)	<0.001 *
Total Number of Brain Areas with Lesions:			
0	5 (9%)	14 (47%)	<0.001 *
1	52 (91%)	13 (43%)	
2	0 (0%)	1 (3%)	
3	0 (0%)	2 (7%)	
Number of Subcortical White Matter Lesions:			
0	10 (18%)	16 (53%)	
1	30 (53%)	13 (43%)	0.001 *
2	11 (19%)	1 (3%)	
3	6 (11%)	0 (0%)	
Number of Deep White Matter Lesions:			
0	25 (44%)	24 (80%)	
1	21 (37%)	4 (13%)	0.014 *
2	10 (18%)	2 (7%)	
3	1 (2%)	0 (0%)	
Number of Periventricular White Matter Lesions:			
0	37 (65%)	26 (87%)	
1	16 (28%)	4 (13%)	0.073
2	4 (7%)	0 (0%)	
Number of Basal Ganglia Lesions:			
0	54 (95%)	29 (97%)	
1	2 (4%)	1 (3%)	0.77
2	1 (2%)	0 (0%)	
Number of Cortical Lesions:			
0	57 (100%)	29 (97%)	0.17
1	0 (0%)	1 (3%)	
Number of Pontine Lesions:			
0	54 (96%)	30 (100%)	
1	1 (2%)	0 (0%)	0.58
2	1 (2%)	0 (0%)	
Number of Brainstem Lesions:			
0	56 (98%)	28 (97%)	0.62
1	1 (2%)	1 (3%)	
Mesial Temporal Sclerosis:			
0	53 (95%)	28 (97%)	0.69
1	3 (5%)	1 (3%)	

ARD autoimmune rheumatic disease; LV-/RV left/right ventricular; EDV/ESV end-diastolic/-systolic volume; EF ejection fraction; EGE/LGE early/late gadolinium enhancement; ECV extracellular volume fraction; CMR cardiovascular magnetic resonance. * *p* ≤ 0.05.

**Table 2:** Baseline characteristics compared between patients with and without identified brain lesions.

Variable	No Brain Lesions	Brain Lesions	*p*-Value
Number of Patients	19	68	N/A
Demographics			
Age	44.7 (15.4)	48.1 (13.2)	0.35
Female sex	4 (21%)	43 (63%)	0.001 *
ARD patients	5 (26%)	52 (76%)	<0.001 *
Cardiac functional indices			
LVEDV (mL)	134.0 (116.0, 201.0)	135.5 (104.0, 160.0)	0.32
LVESV (mL)	50.0 (43.0, 60.0)	50.0 (36.5, 63.5)	0.55
LVEF (%)	63.0 (56.0, 69.0)	62.0 (60.0, 67.5)	0.96
RVEDV (mL)	121.0 (108.0, 151.0)	122.5 (98.0, 150.0)	0.6
RVESV (mL)	45.0 (32.0, 60.0)	44.0 (35.0, 58.0)	0.95
RVEF (%)	62.0 (58.0, 67.0)	63.0 (60.0, 66.0)	0.82
Tissue characterization indices			
T2 signal ratio	2.1 (1.7, 2.2)	2.0 (1.8, 2.3)	0.75
EGE (%)	2.8 (1.6, 4.4)	2.5 (1.6, 3.0)	0.36
LGE (%)	5.0 (0.0, 6.0)	4.0 (0.0, 5.0)	0.1
Native T1-mapping (ms)	1034.0 (1020.0, 1058.0)	1041.0 (1000.5, 1084.5)	0.86
Post-contrast T1-mapping (ms)	420.0 (380.0, 436.0)	387.0 (352.5, 415.5)	0.052
ECV (%)	26.0 (25.0, 29.0)	29.0 (27.0, 32.0)	0.002 *
T2-Mapping (ms)	53.4 (5.8)	54.6 (7.8)	0.53
Local cut-off points for CMR indices			
LGE > 0%	14 (74%)	40 (59%)	0.24
EGE ≥ 4	5 (26%)	12 (18%)	0.4
T2 ratio ≥ 2	13 (68%)	38 (56%)	0.33
T2-mapping > 55 ms	3 (16%)	27 (40%)	0.052
Native T1-mapping > 1050 ms	7 (37%)	28 (41%)	0.73
ECV ≥ 29%	5 (26%)	35 (51%)	0.052
Post-contrast T1-mapping < 350 ms	4 (21%)	15 (22%)	0.93
LVEF < 55%	3 (16%)	10 (15%)	0.91

ARD autoimmune rheumatic disease; LV-/RV left/right ventricular; EDV/ESV end-diastolic/-systolic volume; EF ejection fraction; EGE/LGE early/late gadolinium enhancement; ECV extracellular volume fraction; CMR cardiovascular magnetic resonance. * *p* ≤ 0.05.

**Table 3:** Results of univariable and multivariable logistic regression analysis for predicting the presence of any type of brain lesion. Multivariable corrections were applied for age and sex to each index separately. This was a pooled analysis of ARD patients and disease controls with and without brain involvement.

Variable	Univariable Logistic Regression		Multivariable Logistic Regression	
	OR (95% CI)	*p*-Value	OR (95% CI)	*p*-Value
LVEDV (per 5 mL)	0.98 (0.94–1.03)	0.422	1.01 (0.96–1.06)	0.817
LVESV (per 5 mL)	0.99 (0.93–1.06)	0.844	1.03 (0.95–1.11)	0.493
LVEF (per 5%)	1.01 (0.75–1.35)	0.957	0.90 (0.65–1.25)	0.525
RVEDV (per 5 mL)	0.98 (0.93–1.04)	0.491	1.02 (0.96–1.09)	0.519
RVESV (per 5 mL)	0.99 (0.89–1.10)	0.823	1.07 (0.94–1.21)	0.309
RVEF (per 5%)	0.97 (0.66–1.44)	0.899	0.79 (0.49–1.28)	0.337
T2 signal ratio	0.87 (0.28–2.65)	0.801	0.60 (0.16–2.16)	0.433
EGE (%)	0.98 (0.86–1.11)	0.766	0.95 (0.83–1.09)	0.474
LGE (%)	0.93 (0.83–1.05)	0.27	0.93 (0.81–1.06)	0.254
Native T1-mapping (per 10 ms)	1.01 (0.95–1.09)	0.702	0.94 (0.86–1.02)	0.169
Post-contrast T1-mapping (per 10 ms)	0.93 (0.85–1.03)	0.168	0.94 (0.85–1.05)	0.283
ECV (%)	1.11 (0.98–1.27)	0.103	1.08 (0.96–1.21)	0.197
T2-mapping (ms)	1.02 (0.95–1.10)	0.53	0.98 (0.90–1.06)	0.563
ARD patients (compared with controls)	9.10 (2.84–29.17)	<0.001 *	6.26 (1.74–22.49)	0.005 *

ARD autoimmune rheumatic disease; LV-/RV left/right ventricular; EDV/ESV end-diastolic/-systolic volume; EF ejection fraction; EGE/LGE early/late gadolinium enhancement; ECV extracellular volume fraction. * *p* ≤ 0.05.

## Supplementary Materials

The following are available online at https://www.mdpi.com/2077-0383/9/2/447/s1, Table S1: Results of univariable and multivariable ordered logistic regression analysis for predicting higher numbers of subcortical white matter lesions, Table S2: Results of univariable and multivariable ordered logistic regression analysis for predicting higher numbers of deep white matter lesions, Table S3: Results of univariable and multivariable ordered logistic regression analysis for predicting higher numbers of periventricular white matter lesions.

## References

[1] Laribi S., Mebazaa A. (2014). Cardiohepatic syndrome: Liver injury in decompensated heart failure. Curr. Heart Fail. Rep..

[2] Swirski F.K., Nahrendorf M. (2018). Cardioimmunology: The immune system in cardiac homeostasis and disease. Nat. Rev. Immunol..

[3] Fulton W.F. (1963). Arterial Anastomoses in the Coronary Circulation. I. Anatomical Features in Normal and Diseased Hearts Demonstrated by Stereoarteriography. Scott. Med. J..

[4] Noel Bairey Merz C., Pepine C.J., Walsh M.N., Fleg J.L. (2017). Ischemia and No Obstructive Coronary Artery Disease (INOCA): Developing Evidence-Based Therapies and Research Agenda for the Next Decade. Circulation.

[5] Pantoni L. (2010). Cerebral small vessel disease: From pathogenesis and clinical characteristics to therapeutic challenges. Lancet Neurol..

[6] Conen D., Rodondi N., Müller A., Beer J.H., Ammann P., Moschovitis G., Auricchio A., Hayoz D., Kobza R., Shah D. (2019). Relationships of Overt and Silent Brain Lesions with Cognitive Function in Patients with Atrial Fibrillation. J. Am. Coll. Cardiol..

[7] Haller S., Kövari E., Herrmann F.R., Cuvinciuc V., Tomm A.-M., Zulian G.B., Lovblad K.-O., Giannakopoulos P., Bouras C. (2013). Do brain T2/FLAIR white matter hyperintensities correspond to myelin loss in normal aging? A radiologic-neuropathologic correlation study. Acta Neuropathol. Commun..

[8] Ylikoski A., Erkinjuntti T., Raininko R., Sarna R., Sulkava R., Tilvis R. (1995). White matter hyperintensities on mri in the neurologically nondiseased elderly: Analysis of cohorts of consecutive subjects aged 55 to 85 years living at home. Stroke.

[9] Debette S., Markus H.S. (2010). The clinical importance of white matter hyperintensities on brain magnetic resonance imaging: Systematic review and meta-analysis. BMJ.

[10] Garde E., Mortensen E.L., Krabbe K., Rostrup E., Larsson H.B.W. (2000). Relation between age-related decline in intelligence and cerebral white-matter hyperintensities in healthy octogenarians: A longitudinal study. Lancet.

[11] Nurmohamed M.T., Heslinga M., Kitas G.D. (2015). Cardiovascular comorbidity in rheumatic diseases. Nat. Rev. Rheumatol..

[12] Mavrogeni S.I., Kitas G.D., Dimitroulas T., Sfikakis P.P., Seo P., Gabriel S., Patel A.R., Gargani L., Bombardieri S., Matucci-Cerinic M. (2016). Cardiovascular magnetic resonance in rheumatology: Current status and recommendations for use. Int. J. Cardiol..

[13] Prasad M., Hermann J., Gabriel S.E., Weyand C.M., Mulvagh S., Mankad R., Oh J.K., Matteson E.L., Lerman A. (2015). Cardiorheumatology: Cardiac involvement in systemic rheumatic disease. Nat. Rev. Cardiol..

[14] Guzman-Martinez L., Maccioni R.B., Andrade V., Navarrete L.P., Pastor M.G., Ramos-Escobar N. (2019). Neuroinflammation as a common feature of neurodegenerative disorders. Front. Pharmacol..

[15] DiSabato D.J., Quan N., Godbout J.P. (2016). Neuroinflammation: The devil is in the details. J. Neurochem..

[16] Gerdes V.E.A., Kwa V.I.H., ten Cate H., Brandjes D.P.M., Büller H.R., Stam J. (2006). Cerebral white matter lesions predict both ischemic strokes and myocardial infarctions in patients with established atherosclerotic disease. Atherosclerosis.

[17] Mäntylä R., Aronen H.J., Salonen O., Pohjasvaara T., Korpelainen M., Peltonen T., Standertskjöld-Nordenstam C.G., Kaste M., Erkinjuntti T. (1999). Magnetic resonance imaging white matter hyperintensities and mechanism of ischemic stroke. Stroke.

[18] Jefferson A.L., Tate D.F., Poppas A., Brickman A.M., Paul R.H., Gunstad J., Cohen R.A. (2007). Lower cardiac output is associated with greater white matter hyperintensities in older adults with cardiovascular disease. J. Am. Geriatr. Soc..

[19] Moroni F., Ammirati E., Rocca M.A., Filippi M., Magnoni M., Camici P.G. (2018). Cardiovascular disease and brain health: Focus on white matter hyperintensities. IJC Hear. Vasc..

[20] Wei C., Zhang S., Liu J., Yuan R., Liu M. (2018). Relationship of cardiac biomarkers with white matter hyperintensities in cardioembolic stroke due to atrial fibrillation and/or rheumatic heart disease. Medicine.

[21] Wiseman S.J., Ralston S.H., Wardlaw J.M. (2016). Cerebrovascular disease in rheumatic diseases a systematic review and meta-analysis. Stroke.

[22] Friedrich M.G., Sechtem U., Schulz-Menger J., Holmvang G., Alakija P., Cooper L.T., White J.A., Abdel-Aty H., Gutberlet M., Prasad S. (2009). Cardiovascular Magnetic Resonance in Myocarditis: A JACC White Paper. J. Am. Coll. Cardiol..

[23] Mavrogeni S.I., Sfikakis P.P., Markousis-Mavrogenis G., Bournia V.K., Poulos G., Koutsogeorgopoulou L., Karabela G., Stavropoulos E., Katsifis G., Boki K. (2019). Cardiovascular magnetic resonance imaging pattern in patients with autoimmune rheumatic diseases and ventricular tachycardia with preserved ejection fraction. Int. J. Cardiol..

[24] Mavrogeni S., Kitas G.D., Lamb H.J., Psychoyios K., Dimitroulas T., Koutsogeorgopoulou L., Boki K., Vartela V., Kolovou G., Markousis-Mavrogenis G. (2018). Combined brain and heart magnetic resonance imaging in systemic vasculitides: Fiction or real need?. Clin. Exp. Rheumatol..

[25] Breheny P., Huang J. (2011). Coordinate descent algorithms for nonconvex penalized regression, with applications to biological feature selection. Ann. Appl. Stat..

[26] Miller R.E., Breheny P. (2019). Marginal false discovery rate control for likelihood-based penalized regression models. Biometrical J..

[27] Breheny P.J. (2019). Marginal false discovery rates for penalized regression models. Biostatistics.

[28] Liu H., Du G., Zhang L., Lewis M.M., Wang X., Yao T., Li R., Huang X. (2016). Folded concave penalized learning in identifying multimodal MRI marker for Parkinson’s disease. J. Neurosci. Methods.

[29] Mehta P., Bukov M., Wang C.H., Day A.G.R., Richardson C., Fisher C.K., Schwab D.J. (2019). A high-bias, low-variance introduction to Machine Learning for physicists. Phys. Rep..

[30] Nanea I.T., Gheorghe G.S. (2018). Right ventricular function in systemic autoimmune diseases. Right Heart Pathology: From Mechanism to Management.

[31] Greulich S., Mayr A., Kitterer D., Latus J., Henes J., Steubing H., Kaesemann P., Patrascu A., Greiser A., Groeninger S. (2017). T1 and T2 mapping for evaluation of myocardial involvement in patients with ANCA-associated vasculitides. J. Cardiovasc. Magn. Reson..

[32] Hinojar R., Foote L., Sangle S., Marber M., Mayr M., Carr-White G., D’Cruz D., Nagel E., Puntmann V.O. (2016). Native T1 and T2 mapping by CMR in lupus myocarditis: Disease recognition and response to treatment. Int. J. Cardiol..

[33] Puntmann V.O., Isted A., Hinojar R., Foote L., Carr-White G., Nagel E. (2017). T1 and T2 mapping in recognition of early cardiac involvement in systemic sarcoidosis. Radiology.

[34] Ackermann C., van Toorn R., Andronikou S. (2019). Human immunodeficiency virus-related cerebral white matter disease in children. Pediatr. Radiol..

[35] Soriano-Raya J.J., Miralbell J., López-Cancio E., Bargalló N., Arenillas J.F., Barrios M., Cáceres C., Toran P., Alzamora M., Dávalos A. (2012). Deep versus periventricular white matter lesions and cognitive function in a community sample of middle-aged participants. J. Int. Neuropsychol. Soc..

[36] Degroot A., Treit D. (2002). Dorsal and ventral hippocampal cholinergic systems modulate anxiety in the plus-maze and shock-probe tests. Brain Res..

[37] Price R.W., Swanstrom R. (2012). Targeting chronic central nervous system HIV infection. Antivir. Ther..

[38] Kim K.W., MacFall J.R., Payne M.E. (2008). Classification of White Matter Lesions on Magnetic Resonance Imaging in Elderly Persons. Biol. Psychiatry.

[39] Lin T.M., Chen W.S., Sheu J.J., Chen Y.H., Chen J.H., Chang C.C. (2018). Autoimmune rheumatic diseases increase dementia risk in middle-aged patients: A nationwide cohort study. PLoS ONE.

[40] André R., Cottin V., Saraux J.L., Blaison G., Bienvenu B., Cathebras P., Dhote R., Foucher A., Gil H., Lapoirie J. (2017). Central nervous system involvement in eosinophilic granulomatosis with polyangiitis (Churg-Strauss): Report of 26 patients and review of the literature. Autoimmun. Rev..

[41] González-Suárez I., Arpa J., Ríos-Blanco J.J. (2016). Brain microvasculature involvement in ANCA positive vasculitis. Cerebrovasc. Dis..

[42] Rodrigues M., Galego O., Costa C., Jesus D., Carvalho P., Santiago M., Malcata A., Inês L. (2017). Central nervous system vasculitis in systemic lupus erythematosus: A case series report in a tertiary referral centre. Lupus.

[43] Koçer N., Islak C., Siva A., Saip S., Akman C., Kantarci O., Hamuryudan V. (1999). CNS involvement in Neuro-Behcet syndrome: An MR study. Am. J. Neuroradiol..

[44] Lacomis D. (2011). Neurosarcoidosis. Curr. Neuropharmacol..

[45] MacÊdo P.J.O.M., Da Silveira V.C., Ramos L.T., Nóbrega F.R., Vasconcellos L.F.R. (2016). Isolated central nervous system vasculitis as a manifestation of neurosarcoidosis. J. Stroke Cerebrovasc. Dis..

[46] Mavrogeni S., Markousis-Mavrogenis G., Koutsogeorgopoulou L., Dimitroulas T., Bratis K., Kitas G.D., Sfikakis P., Tektonidou M., Karabela G., Stavropoulos E. (2017). Cardiovascular magnetic resonance imaging pattern at the time of diagnosis of treatment naïve patients with connective tissue diseases. Int. J. Cardiol..

[47] Mavrogeni S., Koutsogeorgopoulou L., Markousis-Mavrogenis G., Bounas A., Tektonidou M., Lliossis S.-N.N.C., Daoussis D., Plastiras S., Karabela G., Stavropoulos E. (2018). Cardiovascular magnetic resonance detects silent heart disease missed by echocardiography in systemic lupus erythematosus. Lupus.

[48] Hollan I., Meroni P.L., Ahearn J.M., Cohen Tervaert J.W., Curran S., Goodyear C.S., Hestad K.A., Kahaleh B., Riggio M., Shields K. (2013). Cardiovascular disease in autoimmune rheumatic diseases. Autoimmun. Rev..

[49] Van Dijk E.J., Prins N.D., Vermeer S.E., Vrooman H.A., Hofman A., Koudstaal P.J., Breteler M.M.B. (2005). C-reactive protein and cerebral small-vessel disease: The Rotterdam scan study. Circulation.

[50] Fornage M., Chiang Y.A., Omeara E.S., Psaty B.M., Reiner A.P., Siscovick D.S., Tracy R.P., Longstreth W.T. (2008). Biomarkers of inflammation and MRI-defined small vessel disease of the brain: The cardiovascular health study. Stroke.

[51] Mavrogeni S.I., Markousis-Mavrogenis G., Karapanagiotou O., Toutouzas K., Argyriou P., Velitsista S., Kanoupakis G., Apostolou D., Hautemann D., Sfikakis P.P. (2019). Silent Myocardial Perfusion Abnormalities Detected by Stress Cardiovascular Magnetic Resonance in Antiphospholipid Syndrome: A Case-Control Study. J. Clin. Med..

[52] Mavrogeni S., Koutsogeorgopoulou L., Karabela G., Stavropoulos E., Katsifis G., Raftakis J., Plastiras S., Noutsias M., Markousis-Mavrogenis G., Kolovou G. (2017). Silent myocarditis in systemic sclerosis detected by cardiovascular magnetic resonance using Lake Louise criteria. BMC Cardiovasc. Disord..

[53] Mavrogeni S.I., Sfikakis P.P., Koutsogeorgopoulou L., Markousis-Mavrogenis G., Dimitroulas T., Kolovou G., Kitas G.D. (2017). Cardiac Tissue Characterization and Imaging in Autoimmune Rheumatic Diseases. JACC Cardiovasc. Imaging.

[54] De Lorenzis E., Gremese E., Bosello S., Nurmohamed M.T., Sinagra G., Ferraccioli G. (2019). Microvascular heart involvement in systemic autoimmune diseases: The purinergic pathway and therapeutic insights from the biology of the diseases. Autoimmun. Rev..

