# Supplementary material for: Combined Brain-Heart Magnetic Resonance Imaging in Autoimmune Rheumatic Disease Patients with Cardiac Symptoms: Hypothesis Generating Insights from a Cross-Sectional Study
Source: J Clin Med. 2020 Feb 6;9(2):447. doi: 10.3390/jcm9020447 (PMC7074384; doi:10.3390/jcm9020447)
Supplement: Supplementary file 1 [file jcm-09-00447-s001.pdf]

Supplementary Table 1. Results of univariable and multivariable ordered logistic regression analysis for predicting higher numbers of subcortical white matter lesions. Multivariable corrections were applied for age and sex.

Variable	Univariable Ordered Logistic Regression		Multivariable Ordered Logistic Regression	
	OR (95% CI)	p-value	OR (95% CI)	p-value
**LVEDV (per 5 mL)**	0.98 (0.94-1.02)	0.318	1.0 (0.96-1.05)	0.984
**LVESV (per 5 mL)**	0.99 (0.94-1.05)	0.785	1.02 (0.96-1.09)	0.466
**LVEF (per 5%)**	1.04 (0.82-1.33)	0.729	0.95 (0.73-1.22)	0.677
**RVEDV (per 5 mL)**	0.97 (0.93-1.02)	0.220	1.00 (0.95-1.06)	0.869
**RVESV (per 5 mL)**	0.96 (0.88-1.04)	0.329	1.02 (0.92-1.12)	0.718
**RVEF (per 5%)**	1.16 (0.84-1.60)	0.378	1.01 (0.73-1.40)	0.960
**T2 Signal Ratio**	1.36 (0.57-3.23)	0.486	0.96 (0.39-2.36)	0.935
**EGE (%)**	0.99 (0.89-1.09)	0.832	0.98 (0.89-1.09)	0.727
**LGE (%)**	1.06 (0.96-1.18)	0.216	1.07 (0.96-1.18)	0.238
**Native T1-Mapping (per 10 ms)**	1.02 (0.97-1.07)	0.437	0.97 (0.92-1.03)	0.313
**Post-contrast T1-Mapping (per 10 ms)**	0.94 (0.88-1.00)	0.057	0.94 (0.88-1.01)	0.113
**ECV (%)**	1.09 (0.99-1.20)	0.081	1.06 (0.97-1.16)	0.206
**T2-Mapping (ms)**	1.02 (0.97-1.08)	0.332	0.99 (0.94-1.05)	0.764
**ARD patients (compared with controls)**	6.25 (2.46-15.90)	**<0.001***	5.02 (1.82-13.84)	**0.002***

ARD autoimmune rheumatic disease; LV- / RV left/right ventricular; EDV/ESV end-diastolic/-systolic volume; EF ejection fraction; EGE/LGE early/late gadolinium enhancement; ECV extracellular volume fraction

Supplementary Table 2. Results of univariable and multivariable ordered logistic regression analysis for predicting higher numbers of deep white matter lesions. Multivariable corrections were applied for age and sex.

Variable	Univariable Ordered Logistic Regression		Multivariable Ordered Logistic Regression	
	OR (95% CI)	p-value	OR (95% CI)	p-value
**LVEDV (per 5 mL)**	1.01 (0.97-1.05)	0.720	1.03 (0.99-1.08)	0.135
**LVESV (per 5 mL)**	1.02 (0.96-1.08)	0.511	1.05 (0.99-1.12)	0.101
**LVEF (per 5%)**	0.90 (0.71-1.14)	0.395	0.83 (0.64-1.06)	0.135
**RVEDV (per 5 mL)**	0.99 (0.94-1.03)	0.553	1.01 (0.96-1.07)	0.622
**RVESV (per 5 mL)**	0.98 (0.90-1.08)	0.693	1.03 (0.93-1.14)	0.553
**RVEF (per 5%)**	1.05 (0.77-1.43)	0.768	0.97 (0.71-1.33)	0.843
**T2 Signal Ratio**	1.08 (0.43-2.68)	0.871	0.79 (0.29-2.15)	0.643
**EGE (%)**	0.92 (0.78-1.08)	0.300	0.92 (0.78-1.08)	0.297
**LGE (%)**	1.09 (0.99-1.20)	0.067	1.09 (0.99-1.21)	0.091
**Native T1-Mapping (per 10 ms)**	1.02 (0.97-1.07)	0.407	0.98 (0.93-1.04)	0.608
**Post-contrast T1-Mapping (per 10 ms)**	0.93 (0.87-0.99)	**0.046***	0.94 (0.87-1.01)	0.073
**ECV (%)**	1.19 (1.04-1.35)	**0.007***	1.16 (1.01-1.33)	**0.031***
**T2-Mapping (ms)**	1.03 (0.98-1.08)	0.310	1.00 (0.95-1.06)	0.916
**ARD patients (compared with controls)**	4.90 (1.76-13.67)	**0.002***	4.69 (1.49-14.74)	**0.008***

ARD autoimmune rheumatic disease; LV- / RV left/right ventricular; EDV/ESV end-diastolic/-systolic volume; EF ejection fraction; EGE/LGE early/late gadolinium enhancement; ECV extracellular volume fraction

Supplementary Table 3. Results of univariable and multivariable ordered logistic regression analysis for predicting higher numbers of periventricular white matter lesions. Multivariable corrections were applied for age and sex.

Variable	Univariable Ordered Logistic Regression		Multivariable Ordered Logistic Regression	
	OR (95% CI)	p-value	OR (95% CI)	p-value
**LVEDV (per 5 mL)**	1.00 (0.96-1.05)	0.895	1.01 (0.96-1.06)	0.708
**LVESV (per 5 mL)**	1.01 (0.95-1.07)	0.770	1.02 (0.96-1.08)	0.608
**LVEF (per 5%)**	1.03 (0.78-1.35)	0.844	0.99 (0.75-1.31)	0.962
**RVEDV (per 5 mL)**	0.99 (0.94-1.04)	0.675	0.99 (0.93-1.05)	0.766
**RVESV (per 5 mL)**	0.97 (0.87-1.07)	0.522	0.97 (0.86-1.09)	0.611
**RVEF (per 5%)**	1.30 (0.88-1.91)	0.193	1.23 (0.83-1.82)	0.298
**T2 Signal Ratio**	0.47 (0.15-1.42)	0.180	0.35 (0.10-1.20)	0.095
**EGE (%)**	0.90 (0.72-1.13)	0.355	0.90 (0.72-1.13)	0.371
**LGE (%)**	1.06 (0.95-1.19)	0.290	1.06 (0.95-1.19)	0.320
**Native T1-Mapping (per 10 ms)**	0.98 (0.92-1.05)	0.580	0.96 (0.90-1.04)	0.305
**Post-contrast T1-Mapping (per 10 ms)**	1.02 (0.94-1.11)	0.579	1.03 (0.95-1.12)	0.455
**ECV (%)**	1.08 (0.95-1.23)	0.251	1.08 (0.94-1.25)	0.277
**T2-Mapping (ms)**	0.99 (0.93-1.06)	0.738	0.98 (0.91-1.05)	0.476
**ARD patients (compared with controls)**	3.63 (1.12-11.86)	**0.032***	4.82 (1.28-18.1)	**0.020***

ARD autoimmune rheumatic disease; LV- / RV left/right ventricular; EDV/ESV end-diastolic/-systolic volume; EF ejection fraction; EGE/LGE early/late gadolinium enhancement; ECV extracellular volume fraction

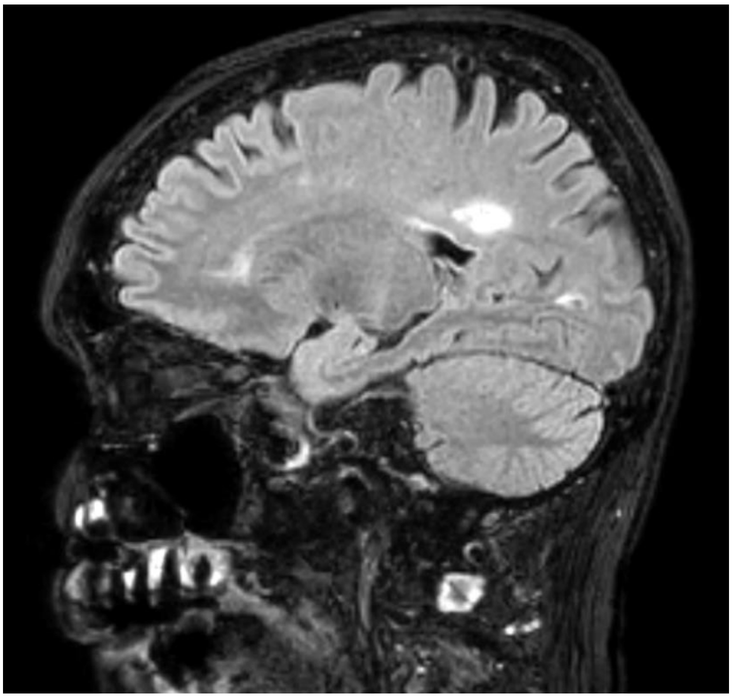

Figure 1. A fluid attenuation inversion recovery (FLAIR) image from a patient with sarcoidosis showing silent white matter hyperintensities.
